# Recurrent terminal ventricle cyst: a case report

**DOI:** 10.1093/jscr/rjab498

**Published:** 2021-11-11

**Authors:** Asfand Baig Mirza, Ravindran Visagan, Timothy Boardman, Christopher Murphy, Bilal Al-Ali, Christopher Kellett, Gordan Grahovac

**Affiliations:** Department of Neurosurgery, King’s College Hospital NHS Foundation Trust, London, UK; Department of Neurosurgery, King’s College Hospital NHS Foundation Trust, London, UK; GKT School of Medical Education, King’s College London, London, UK; Department of Neurosurgery, King’s College Hospital NHS Foundation Trust, London, UK; GKT School of Medical Education, King’s College London, London, UK; Department of Neurosurgery, King’s College Hospital NHS Foundation Trust, London, UK; Department of Neurosurgery, King’s College Hospital NHS Foundation Trust, London, UK

## Abstract

The terminal ventricle (TV) of Krause is a rare cystic dilation of the conus’ central canal. Due to limited understanding surrounding its pathophysiology, optimal management remains controversial. We report a 25-year-old female presenting with acute paraparesis. Magnetic resonance imaging spine revealed a cystic conus medullaris lesion in keeping with an incidental TV cyst. However, the patient experienced a rapid resolution of symptoms. We hypothesize that the TV cyst spontaneously ruptured and auto-decompressed. To our knowledge, this is the first reported case of an enlarging symptomatic TV cyst with spontaneous rupture and resolution of symptoms, highlighting the variable natural history of this condition.

## INTRODUCTION

The terminal ventricle (TV) is a dilation of the conus’ central canal, lined by ependymal cells containing CSF. It was first reported by Stilling in 1859, termed the fifth ventricle by Krause in 1875 and is formed during embryogenesis at the caudal aspect of the central canal and is thought to regress in early childhood [1, 2]. Kernohan’s original post-mortem studies observed VT in all foetuses and children, more than adults with a second relative incidence in the elderly [3]. It has been reported in up to 2.6% children less than 5 years old in a retrospective study of 418 patients, with a female to male ratio of 0.8:1.0 [4]. The adult incidence of TV remains uncertain.

It is clinically important to differentiate between a TV and TV cyst: TV is a persistent, typically asymptomatic anatomical variant of little clinical significance. TV/conus medullaris cyst is an abnormal cystic dilation, which may progress leading to neurological symptoms.

The natural history of TV cysts is poorly understood with some reports suggesting a benign non-progressive course and others describing a more hazardous natural history with cyst enlargement and consequent conus medullaris syndrome [5, 7–9].

The authors present a case of acute neurological decline secondary to progressive dilatation of a TV cyst with spontaneous rupture and resolution of symptoms captured with magnetic resonance imaging (MRI). The pathogenesis of enlarging symptomatic TV cysts is hypothesized and presented.

## CASE REPORT

A 25-year-old female presented with a three week history of lower back pain, headache, photophobia and sudden onset paraparesis. On examination, she had 3/5 power in all myotomes of the lower limb with subjective paraesthesia below the knees (L3 dermatome) bilaterally. There was no acute sphincteric, bladder, bowel or perineal sensory dysfunction. She had presented 10 years previously with similar symptoms with spontaneous resolution.

**Figure 1:**
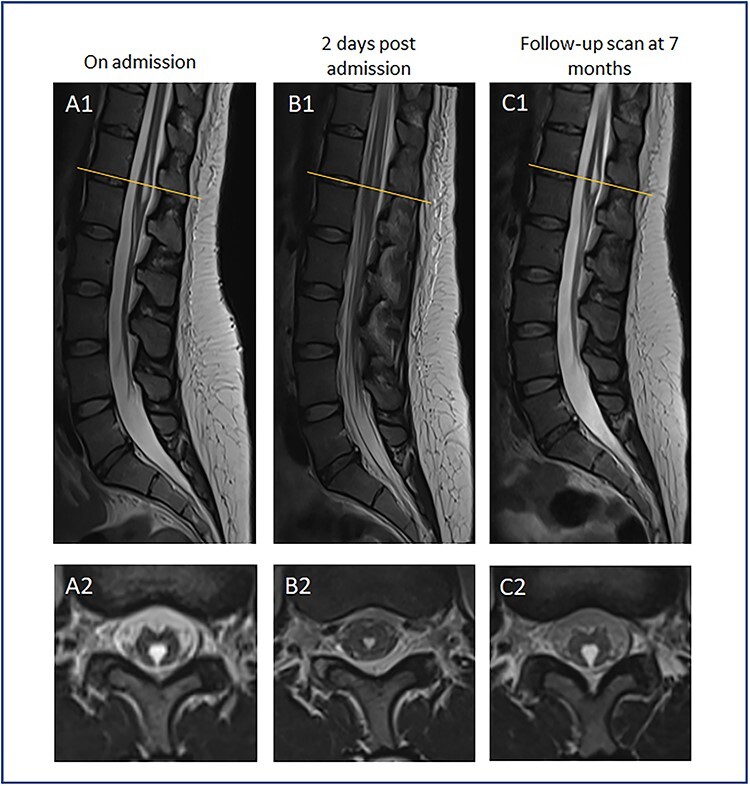
MR spinal imaging during admission and at follow-up. T2 weighted MR sagittal (A1-C1) and axial (A2-C2) images at T12/L1 level (orange line). **A**—Admission MRI, **B**—MRI 2 days post admission, **C**—7 month follow-up scan. A1 + 2 at initial presentation with expanded TV cyst compressing conus causing acute neurological deficit. B1 + 2 showing conus appearances post TV cyst rupture corresponding with resolution of neurological symptoms. C1 + 2 images at 7 month follow-up in keeping with cyst recollection with no corresponding neurological deficit.

Neurological consultation was sought, and lumbar puncture was performed which revealed a normal cerebrospinal fluid (CSF) profile. On Day 4 of admission she was re-reviewed by the neurologists where she was found to have no movement in her lower limbs (0/5 power). Her lower limb reflexes were present bilaterally with downgoing plantar responses. A whole spine MRI was performed which revealed a TV cyst (Fig. 1). On Day 6, she reported complete resolution of her symptoms with a normal neurological examination. A repeat whole spine MRI was performed which showed spontaneous resolution of the cyst in keeping with cyst rupture. The MRI was deemed normal with no evidence of intracranial pathology as reported by the neuroradiologist.

We can only hypothesize rather than prove that the cyst was the cause of this patient’s neurological deficit. The transient neurological signs correlate with the transient results on imaging, whereby the cyst ruptured and later re-expanded again, as discussed below. Neurologist advice was sought, and no other abnormalities in the neuro-axis were found. Hoover’s sign was also negative.

She underwent a short period of inpatient physiotherapy and was discharged home. The patient did not attend further outpatient follow-up. Seven months later she attended her local emergency department with symptoms of bilateral non-dermatomal leg paraesthesia. Lumbo-sacral MRI demonstrated modest enlargement of the TV cyst in keeping with recollection. In the absence of a progressive motor or localizing neurological deficit, surgery was not felt to be indicated and she remains under outpatient surveillance.

## DISCUSSION

This is the first report of symptomatic enlargement of a TV cyst with spontaneous rupture and subsequent recollection. The case offers an insight into the condition’s pathogenesis and natural history.

### Natural History and Clinical Presentation

The TV cyst’s natural history remains poorly understood and it is unknown why a small number undergoes progressive and symptomatic cystic dilation. Symptomatic cases in the literature have been managed surgically and the follow-up of conservatively managed asymptomatic patients is limited. The longest reported follow-up is 10 years during which there was an increase in cyst volume from 1.5 to 3.2mls [10]. Patients can present with chronic asymptomatic progression over years or with acute neurological deterioration [5, 7–9]. Serial MRI can show slow progression of the cyst without any associated conus oedema, while intraoperative findings generally reveal normal CSF contents which can appear to be under pressure [6, 11].

### Proposed hypothesis of TV cyst enlargement and spontaneous rupture

We propose, based on our case, there is a loss of communication of the TV cyst with the central canal at some stage in early adult life. Persistent TV implies obstruction of physiological CSF flow which could be due to ependymal cell growth/death, cellular debris thus proteinaceous build-up increasing relative oncotic pressure. This compressive effect on the conus medullaris leads to neurological symptoms. However, once the intracystic pressure exceeds the transmural pressure of the cyst wall within a closed intraspinal compartment surrounded by ligamentous and bony structures, the cyst ruptures. Therefore, there is an acute relief of compression on the conus and symptom resolution. Although spontaneous rupture may decompress the cyst, this may be a transient phenomenon supported by our serial imaging findings. This supports surgical marsupialization for persistently symptomatic cases with excision of the cyst wall to prevent recurrence.

Interestingly, the patient reported associated meningism prior to rupture as evidenced by the first MRI scan. We hypothesize that sudden cyst expansion/dissection of neural tissue may have potentially caused inflammation and/or vascular injury leading to meningism. Lumbar puncture samples were not specifically examined for CSF bilirubin. Previous cases where intraoperative samples have been taken for fluid analysis revealed CSF with no detectable external constituents.

### Clinical Classification: CLVT

In view of the spectrum of clinical presentation, in an attempt to guide management, de Moura Batista *et al*. [13] offered a clinical classification in 2008: Cystic Lesion of the Ventriculus Terminalis (CLVT) [12], revised in 2012 by Ganau *et al*. [14]. This defines CLVT type Ia (stable non-specific symptoms without clear relation to ventriculus terminalis), type Ib (non-specific but progressive symptoms), type II (focal neurological deficits) and type III (sphincter disturbances). The system advocates conservative treatment of that type Ia VT and surgery for the other types.

## CONCLUSION

This is the first reported case of a symptomatic enlarging TV cyst with spontaneous rupture and clinical improvement. This report offers new insight into the pathogenesis and natural history of TV cysts. We believe initial surveillance in the absence of neurology is safe and marsupialization is mandated in those with progressive neurology and should be considered in recurrent episodes associated with significant neurological decline.

## DISCLOSURE

The authors report no conflict of interest concerning the materials or methods used in this study or the findings specified in this paper.

## CONFLICT OF INTEREST STATEMENT

All authors certify that they have no affiliations with or involvement in any organization or entity with any financial interest (such as honoraria; educational grants; participation in speakers’ bureaus; membership, employment, consultancies, stock ownership, or other equity interest; and expert testimony or patent-licensing arrangements), or non-financial interest (such as personal or professional relationships, affiliations, knowledge or beliefs) in the subject matter or materials discussed in this manuscript.

## FUNDING

No funding was received for this research.

## ETHICAL APPROVAL

All procedures performed in studies involving human participants were in accordance with the ethical standards of the institutional and/or national research committee (name of institute/committee) and with the 1964 Helsinki declaration and its later amendments or comparable ethical standards.

The patient has consented to the submission of the case report for submission to the journal.
